# Draft Genome Sequence of *Lawsonia intracellularis* Strain E40504, Isolated from a Horse Diagnosed with Equine Proliferative Enteropathy

**DOI:** 10.1128/genomeA.00330-17

**Published:** 2017-05-11

**Authors:** Nandita S. Mirajkar, Molly R. Kelley, Connie J. Gebhart

**Affiliations:** a Department of Veterinary and Biomedical Sciences, College of Veterinary Medicine, University of Minnesota, St. Paul, Minnesota, USA; b Veterinary Diagnostic Laboratory, College of Veterinary Medicine, University of Minnesota, St. Paul, Minnesota, USA

## Abstract

Reported herein is the draft genome sequence of equine-origin *Lawsonia intracellularis* strain E40504, an obligate intracellular bacterium and the etiological agent of equine proliferative enteropathy. The 1.69-Mb draft genome sequence includes 1,380 protein-coding genes and 49 RNA genes, and it lacks a genomic island reported in swine-origin *L. intracellularis* strain PHE/MN1-00.

## GENOME ANNOUNCEMENT

Equine proliferative enteropathy (EPE), an emerging disease affecting primarily weaned foals and, occasionally, adult horses, is characterized by anorexia, fever, weight loss, colic, diarrhea, peripheral edema, and hypoproteinemia. *Lawsonia intracellularis*, the etiologic agent of EPE, is an obligate intracellular, motile, and Gram-negative bacterium (1). *L. intracellularis* is also an economically significant pig pathogen and is known to infect a wide range of domestic and wild animals (2). Despite the increasing interest in and occurrence of EPE, there are no publicly available genomes of equine-origin *L. intracellularis*. To investigate EPE pathogenesis and the genomic differences between strains isolated from different host species, we sequenced the genome of equine-origin *L. intracellularis* strain E40504.

DNA was extracted from supernatants of infected McCoy cell cultures using the DNeasy blood and tissue kit (Qiagen, Valencia, CA, USA), as per the manufacturer’s instructions. The genome was sequenced using Sequencing-by-Synthesis chemistry at the University of Minnesota Genomics Center, MN, USA. Briefly, the DNA library was size-selected using Caliper XT (PerkinElmer, MA, USA) and prepared using the TruSeq DNA sample preparation kit (Illumina, CA, USA), as per the manufacturers’ instructions. Sequencing was carried out using the HiSeq sequencing kit (Illumina) with a paired-end 2 × 100-bp construct on the HiSeq sequencing system (Illumina). This yielded 10,000,645 reads, which corresponded to an average genome coverage of approximately 1,163×. Using the default parameters of the *de novo* assembly tool and the Map Reads to Reference tool of CLC Genomics Workbench 8.0.2, the reads were assembled by the *de novo* method and the reference (*L. intracellularis* strain PHE/MN1-00)-guided method, respectively. The nonmapped reads were analyzed by BLAST to remove any potential eukaryotic contamination. Filters were applied to extract a subset of contigs with consensus length ≥1 kb and coverage ≥50×, which were then analyzed by BLAST to confirm the absence of any eukaryotic DNA. The resulting E40504 draft genome was annotated using NCBI Prokaryotic Genome Annotation Pipeline (3). MAUVE (4) was used to visualize regions of difference, and the EZBioCloud average nucleotide identity (ANI) calculator (5) was used to determine the ANI between equine- and swine-origin strains.

The draft genome of equine-origin *L. intracellularis* strain E40504 comprised 33 contigs corresponding to 1,692,973 bp and a G+C content of 32.9%. The 1,456 genes identified included 1,380 protein-coding genes, 49 RNA genes (42 tRNAs, 3 rRNAs, and 4 noncoding RNAs [ncRNAs]), and 27 pseudogenes. Compared with swine-origin strain PHE/MN1-00, the equine-origin strain E40504 showed 99.63% nucleotide identity. The alignment of the genomes suggests that, similar to the swine-origin strain, this equine-origin strain possibly also contains multiple plasmids. The 18-kb prophage-associated genomic island previously described in low-passage-number swine-origin *L. intracellularis* strains (6) was confirmed to be absent in this equine-origin strain E40504. It has been suggested that this genomic island may have contributed to the ecological specialization of this pathogen for the swine host species (6).

### Accession number(s).

This draft genome sequence has been deposited in DDBJ/ENA/GenBank under the accession number MTPJ00000000. The version described in this paper is the first version.
